# Chamber identity programs drive early functional partitioning of the heart

**DOI:** 10.1038/ncomms9146

**Published:** 2015-08-26

**Authors:** Christian Mosimann, Daniela Panáková, Andreas A. Werdich, Gabriel Musso, Alexa Burger, Katy L. Lawson, Logan A. Carr, Kathleen R. Nevis, M. Khaled Sabeh, Yi Zhou, Alan J. Davidson, Anthony DiBiase, Caroline E. Burns, C. Geoffrey Burns, Calum A. MacRae, Leonard I. Zon

**Affiliations:** 1 Howard Hughes Medical Institute, Boston, Massachusetts 02115, USA; 2 Stem Cell Program, Boston Children's Hospital, Boston, Massachusetts 02115, USA; 3 Division of Hematology/Oncology, Boston Children's Hospital, Harvard Stem Cell Institute, Harvard Medical School, Boston, Massachusetts 02115, USA; 4 Institute of Molecular Life Sciences (IMLS), University of Zürich, 8057 Zürich, Switzerland; 5 Cardiovascular Division, Brigham and Women's Hospital, Harvard Medical School, Boston, Massachusetts 02115, USA; 6 Cardiovascular Research Center, Massachusetts General Hospital, Harvard Medical School, Charlestown, Massachusetts 02129, USA

## Abstract

The vertebrate heart muscle (myocardium) develops from the first heart field (FHF) and expands by adding second heart field (SHF) cells. While both lineages exist already in teleosts, the primordial contributions of FHF and SHF to heart structure and function remain incompletely understood. Here we delineate the functional contribution of the FHF and SHF to the zebrafish heart using the *cis*-regulatory elements of the *draculin* (*drl*) gene. The *drl* reporters initially delineate the lateral plate mesoderm, including heart progenitors. Subsequent myocardial *drl* reporter expression restricts to FHF descendants. We harnessed this unique feature to uncover that loss of *tbx5a* and *pitx2* affect relative FHF versus SHF contributions to the heart. High-resolution physiology reveals distinctive electrical properties of each heart field territory that define a functional boundary within the single zebrafish ventricle. Our data establish that the transcriptional program driving cardiac septation regulates physiologic ventricle partitioning, which successively provides mechanical advantages of sequential contraction.

## Abstract (web summary)

The heart forms from combining the first with the second heart field, which in mammals creates left and right ventricle. Here transgenic zebrafish and physiology studies reveal that transcription factors controlling septation in mammals already in teleosts guide muscle coupling by controlling the relative contribution of the two fields to the heart.

## Graphical abstract

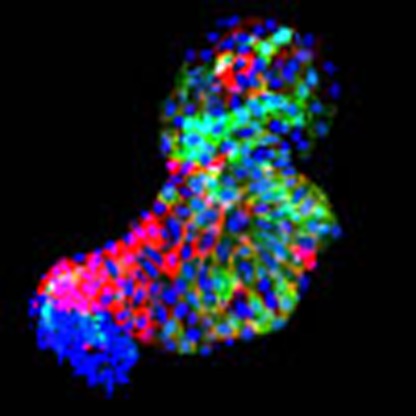

The cardiovascular and haematopoietic lineages arise as multipotent cells in seemingly dispersed territories of the embryo. Shared FLK1 expression^1^ and common regulation by the transcription factor SCL/TAL1 (ref. 2) suggest that the synchronous development of the cardiovascular and haematopoietic lineages results either from exposure to similar signalling environments in different parts of the embryo or prior shared specification during gastrulation. Similarly, OSR1 and HAND2 broadly demarcate bilateral areas within mesoderm with putative cardiac, endothelial, and renal potential^3^, while the adjoining precursors for cardiac muscle and upper limbs express the T-box transcription factor TBX5 (ref. 4). The lateral plate mesoderm thus provides a molecular framework for remarkably distinct, yet intimately connected developmental cell lineages.

The heart forms as a primitive tube derived from cells migrating in the anterior lateral plate mesoderm (ALPM) that have been characterized as the first heart field (FHF), before elongating through the addition of second heart field (SHF) cells at either pole^5^,^6^. In higher organisms, the FHF establishes the left ventricle that supports systemic circulation, while the SHF forms the atria and the right ventricle that is central to pulmonary circulation as well as contributing to the outflow tracts. This division, demarcated by a physical interventricular septum, has suggested a later evolutionary origin of the SHF as a part of the terrestrial vertebrate body plan with a dedicated lung circuit. Recent findings contest this hypothesis, as the teleost zebrafish (*Danio rerio*)^7^,^8^,^9^,^10^ and even the tunicate Ciona (*Ciona intestinalis*)^11^ also feature SHF myocardium, revealing an ancient evolutionary origin of the two heart field lineages before the emergence of septation and pulmonary circulation^12^. The factors determining the emergence of this complex divide into FHF and SHF remain elusive as indeed do the factors that control how these cellular populations migrate and integrate into the maturing organ. This uncertainty is in part due to the limited number of genetic FHF markers^13^,^14^,^15^, the incompletely delineated developmental relationships of the two myocardial lineages, and the limited data on their distinct functional repertoires.

Key regulators of septum formation between the left and right ventricle in mammals, including the transcription factors TXB5 and PITX2, feature loss-of-function phenotypes that affect development of the two-chambered zebrafish heart^4^,^16^,^17^, suggesting uncharacterized primordial functions of these septation regulators before the evolution of any physical boundaries between heart chambers. Here we used transgenic zebrafish reporters based on the regulatory element of the draculin (*drl*) gene to investigate the interplay between the two heart fields. We find that the septation regulators *tbx5* and *pitx2* control the relative contributions of FHF versus SHF to the heart tube, and that a correct ratio of cells with each of these two fates and the establishment of a precise physiologic boundary between these two populations are necessary for normal cardiac function. Our findings reveal that the fundamental regulatory programs that drive septation in higher vertebrates already coordinate regional myocardial coupling within the teleost ventricle.

## Results

### A zebrafish *drl* reporter labels the lateral plate mesoderm

**Figure 1:**
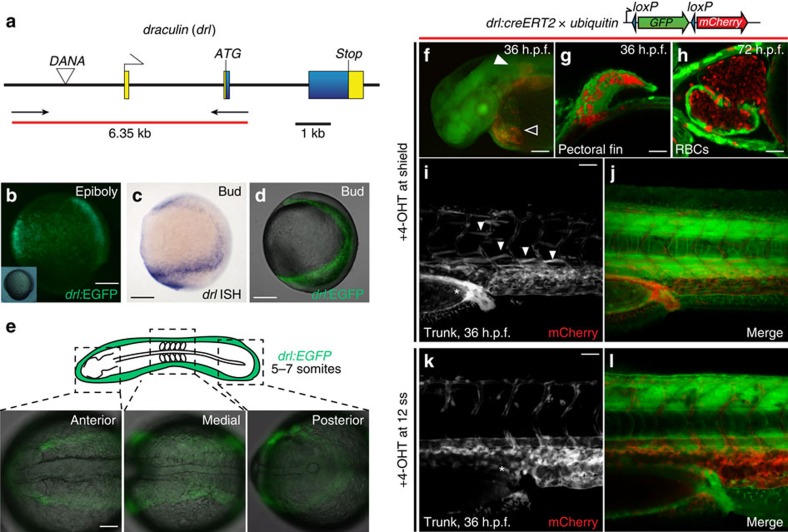
*drl* transgene reporter expression in the early lateral plate mesoderm and cardiovascular development. (**a**) The zebrafish *draculin* (*drl*) locus; blue depicts coding exons (with *ATG* and *Stop* codons), yellow are untranslated regions, a *DANA* retro-transposon is located in the upstream region, arrows and red bar indicate PCR product used for cloning the regulatory sequence. (**b**) *drl:EGFP* reporter expression during gastrulation (80% epiboly, inset shows the embryo in bright-field view), dorsal to the right; scale bar, 100 μm; note the scattered band of cells that form the precursors of the lateral plate mesoderm. (**c**,**d**) Bud stage embryos probed for endogenous *drl* (**c**, mRNA *in situ* hybridization, ISH) versus *drl:EGFP* (**d**, composite bright-field and EGFP confocal stack image) depicting the continuous lateral plate mesoderm; anterior to the top, posterior to the bottom and dorsal to the right; scale bar, 100 μm. (**e**) EGFP fluorescence from homozygous *drl:EGFP* transgenic zebrafish; 5–7 somite stage view of the converging lateral plate mesoderm; scale bar, 100 μm. (**f**–**l**) Genetic lineage tracing using *drl:creERT2* × *ubi:Switch* (schematic on top), 4-OHT-induced at shield stage, labels the lateral plate mesoderm descendants at 36 h.p.f., including cardiac (outlined arrowhead in **f**; scale bar, 250 μm), pectoral fin mesoderm (solid arrowhead in **f**,**g**; scale bar, 50 μm), red blood cells (RBCs, **h**; scale bar, 50 μm), kidney (asterisks in **i**,**j**; scale bar, 50 μm), plus vasculature and scattered superficial trunk muscle cells (solid arrowheads in **i**). (**k**,**l**) 4-OHT induction at 12 somite stage and imaged at 36 h.p.f. refines tracing exclusively to the circulatory system and blood, note absence of kidney tracing (asterisks in **k**); scale bar, 50 μm.

While seeking genetic labels to study the early emergence of haematopoietic and cardiovascular lineages in zebrafish, we isolated the regulatory region of the *drl* gene (Fig. 1a). *drl* encodes a zinc-finger protein of unidentified function, yet is (by messenger RNA (mRNA) *in situ* hybridization (ISH)) the earliest haematopoietic marker to delineate the anterior and posterior haematopoietic territories before becoming undetectable after 24 h.p.f. (hours post fertilization)^18^ (Supplementary Fig. 1). Unlike other blood marker genes, *drl* expression already initiates during gastrulation and condenses as a band of cells at the prospective lateral embryo margin (Fig. 1c; Supplementary Fig. 1). *drl:EGFP* transgenics recapitulate the *drl* ISH pattern: in late epiboly, *drl:EGFP* is detectable as a band of scattered enhanced green fluorescent protein (EGFP)-fluorescent cells (Fig. 1b); after gastrulation, the *drl:EGFP*-positive cells coalesce at the embryo margin (Fig. 1c-e) that then in somitogenesis breaks down into the anterior and posterior lateral plate with subsequent cell migrations that form the posterior vascular/haematopoietic stripes and the anterior cardiovascular and myeloid precursors.

Using zebrafish transgenic for 4-OH-tamoxifen (4-OHT)-inducible *drl:creERT2* crossed to ubiquitous or lineage-specific *loxP* lineage tracers (Fig. 1f–l; Supplementary Fig. 2), we genetically tracked the cell fates of the *drl*-expressing cells. 4-OHT induction of *drl:creERT2* during gastrulation at shield stage (6 h.p.f.) selectively labelled the proposed lateral plate mesoderm-derived organs, including the heart, pectoral fin mesenchyme, blood, endothelium and the kidney (Fig. 1f–l; Supplementary Fig. 2e–j). Transcriptome analysis of FACS-isolated *drl:EGFP*-expressing cells at the two somite stage corroborated our conclusion that *drl* marks the entire early lateral plate mesoderm, as over 90% of fivefold enriched genes (*n*=75) and 17 of 19 uncharacterized genes are transcribed in one or more of the lateral-derived organs (Supplementary Fig. 3). Consistent with the described refinement of *drl* expression to anterior and posterior territories during somitogenesis^18^, 4-OHT induction of *drl:creERT2* during mid-somitogenesis (12 somite stage, 15 h.p.f.) confined labelling to cardiovascular lineages, including haematopoietic stem cell precursors (Fig. 1k,l; Supplementary Fig. 2k,l). These results establish that our isolated *drl* regulatory element recapitulates *drl* expression and for the first time reveals pan-lateral plate mesoderm expression of *drl* during gastrulation, before expression is confined to cardiovascular lineages.

### Myocardial *drl* expression restricts to FHF descendants

**Figure 2:**
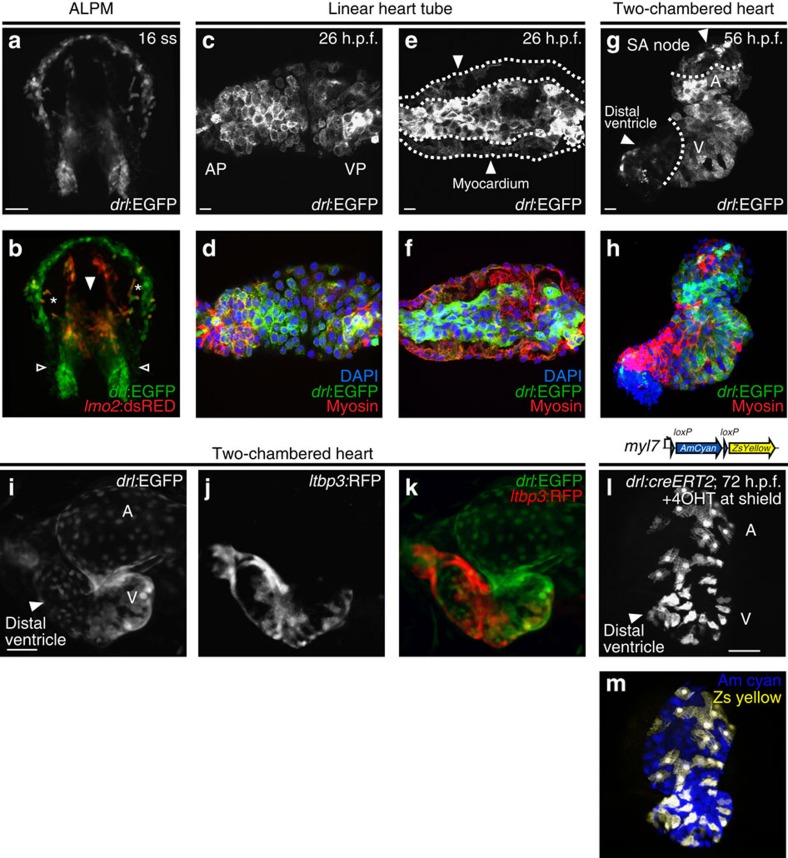
*drl* transgenic reporters track cardiac development and distinguish FHF from SHF myocardium. (**a**,**b**) Confocal projection of the anterior lateral plate mesoderm (ALPM) with hatching gland most anteriorly at 16 somite stage (ss) marked by *drl:EGFP*, dorsal view (**a**, green in **b**) and *lmo2:dsRED2* (endothelial and haematopoietic precursors in red, **b**); asterisks in **b** indicate migrating myeloid precursors, solid arrowhead indicates merging endocardium precursors, and open arrowheads indicate positions of presumptive myocardium precursors; anterior to the top and posterior to the bottom; scale bar, 100 μm. (**c**–**f**) Confocal projections of the linear heart tube at 26 h.p.f., anterior to the left, arterial pole (AP) and venous pole (VP) indicated, (**c**,**d**) top-down 2-μm projection, (**e**,**f**) transversal optical 2-μm section through heart tube. *drl* expression (*drl:EGFP* counter-stained with anti-GFP) in both the central endocardium and the myocardium (counter stained with anti-myosin/MF20 in red, and 4,6-diamidino-2-phenylindole (DAPI) in blue for nuclei in **d**,**f**; scale bar, 10 μm. (**g**,**h**) 56 h.p.f. two-chambered hearts transgenic for *drl*:EGFP top-down 2-μm confocal projection counter stained for anti-GFP (**g**,**h**), anti-myosin/MF20 in red, DAPI in blue for nuclei in **h**. EGFP is expressed in outer ventricular curvature and part of atria, with dotted lines indicating the margins of expression domains, the solid arrowhead point to *drl*:EGFP-negative regions at sinoatrial node (SA) and distal ventricle including outflow tract in **g**; scale bar, 10 μm. (**i**–**k**) Ventral view of a two-chambered heart from a *drl:EGFP; ltbp3:TagRFP2Acre* double-transgenic embryo at 72 h.p.f.; at this stage, *drl:EGFP*-positive FHF-derived cardiac cells (**i**, green in **k**) comprise central portion of the cardiac tube, while the SHF-derived distal ventricular cardiomyocytes population (arrowhead in **i**) is labelled by the *ltbp3:TagRFP2Acre* transgene (**j**, red in **k**); scale bar, 50 μm. (**l**,**m**) *drl:creERT2* × *myl7:loxP-Amcyan-loxP-ZsYellow* (schematic) induced at shield stage labels all myocardial descendants at 72 h.p.f. including the outflow tract cardiomyocytes in the distal ventricle (arrowhead, **l**); scale bar, 50 μm. Atrium (A) and ventricle (V) labelled in **g**,**i**,**l**.

The anterior *drl:EGFP* reporter expression domain in later somitogenesis encompasses the *lmo2*-positive precursors for cranial endothelium, primitive macrophages, and endocardium, as well as *drl*-positive, *lmo2*-negative bilateral foci in the location of cardiomyocyte and pectoral fin precursors^19^,^20^ (Fig. 2a,b). *drl:EGFP* reporter expression is thus seemingly broader than endogenous *drl* expression, which in the ALPM is traditionally attributed to myeloid precursors^18^. Curiously, while endogenous *drl* expression becomes undetectable by ISH post 24 h.p.f. (ref. 18), *drl:EGFP* remained active. This extended *drl:EGFP* reporter expression prompted us to investigate its dynamics during heart formation. Wild-type embryos expressed *drl:EGFP* at 26 h.p.f. in the entire linear heart tube, both endocardium and myocardium (Fig. 2c–f). In *drl:EGFP* hearts at 56 h.p.f., stained for EGFP and myosin, we found that the cardiomyocytes of the distal ventricle, including the outflow tract, as well as those of the proximal atrium were *drl:EGFP* negative, while the central portion of the heart tube and endocardium remained *drl:EGFP* positive (Fig. 2g,h; Supplementary Fig. 4). These observations suggests that late *drl* reporter expression in the myocardium might selectively mark the FHF structures^7^,^9^,^10^.

Consistent with FHF-specific restriction of *drl* reporter expression, *ltbp3* demarcates cardiomyocytes in the distal ventricle and outflow tract of the developing zebrafish heart, including but not restricted to the ventricular SHF population^7^ that is *drl* negative in *drl:EGFP*; *ltbp3:TagRFP* double-transgenic hearts (Fig. 2i–k). Of note, the *drl* negative cardiomyocytes of the sinoatrial junction are also negative for *ltbp3* (ref. 7), but also arise from SHF in zebrafish^10^,^21^ and mammals^22^. Both the FHF and SHF derive from the early, *drl*-expressing lateral plate mesoderm, as *drl:creERT2* induced during gastrulation lineage-labelled all myocardium (Fig. 2l,m). Taken together, these findings propose the *drl*:*EGFP* reporter as a marker of the FHF myocardium after the linear heart tube stage, and extend previous findings in zebrafish by showing that both major myocardial lineages derive initially from the lateral plate mesoderm. *drl:EGFP* thus establishes a genetic means to investigate the relative contributions of FHF and SHF to the heart.

### SHF addition to the heart also occurs without endocardium

**Figure 3:**
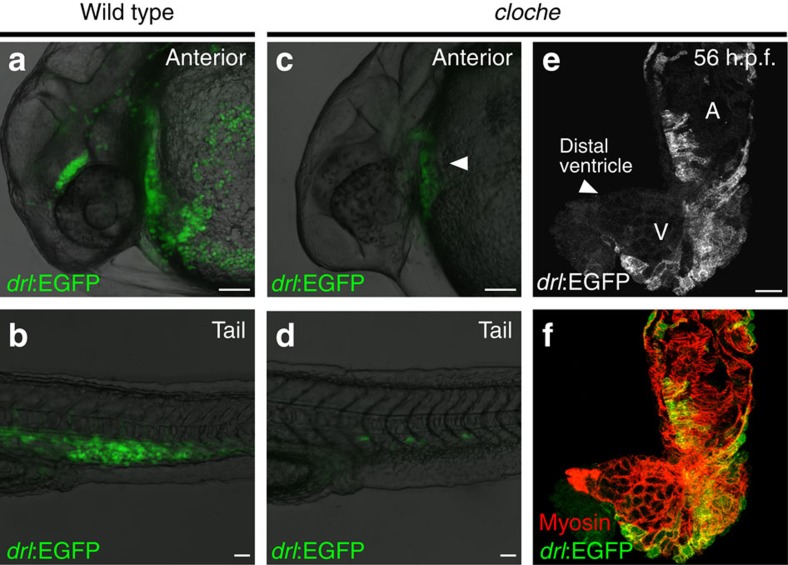
Lack of endocardial signals does not affect integration of FHF with SHF. (**a**,**b**) Anterior (**a**) and tail region (**b**) expression of *drl:EGFP* in wild-type zebrafish at 24–28 h.p.f., scale bar, 50 μm; note the circulating red blood cells. (**c**,**d**) Anterior (**c**) and tail region (**d**) *drl:EGFP* expression at 24–28 h.p.f. in the recessive zebrafish mutant *cloche*, which lacks all endothelial and haematopoietic lineages; scale bar, 50 μm; note the formation of linear heart tube in *cloche* (solid arrowhead in **c** and residual angioblasts in the tail (**d**). (**e**,**f**) Top-down 2-μm confocal projection of 56 h.p.f. *cloche* mutant heart, *drl:EGFP* expression counter stained with anti-GFP (**e**, green in **f**) and cardiomyocytes stained with myosin/MF20 (red in **f**); scale bar, 50 μm; note how despite missing endocardium the heart features both demarcated FHF and SHF myocardium, solid arrowhead in **e** indicates the distal ventricle region. Atrium (A) and ventricle (V) labelled in **e**.

As *drl* reporter expression remains active in the endocardium and in circulating red blood cells (Fig. 3a,b), we sought to genetically isolate the myocardial expression of our *drl* reporter. *drl:EGFP* in zebrafish mutant for *cloche*, which has been well-established to lack all haematopoietic and endothelial lineages from early somitogenesis onward^23^, also labelled the beating linear heart tube at 26 h.p.f. and the few previously reported persisting angioblast precursors in the tail^23^ (Fig. 3c,d).

At 56 h.p.f., *cloche* mutant hearts featured both *drl*-positive FHF and *drl*-negative SHF myocardium territories that did not mix despite the invariant absence of endocardial tissue (Fig. 3e,f). This observation suggests that the addition of SHF cardiomyocytes to the linear heart tube is independent of endocardial signals and can occur in the absence of endothelial and haematopoietic lineages.

### Septation program genes influence FHF and SHF contributions

**Figure 4:**
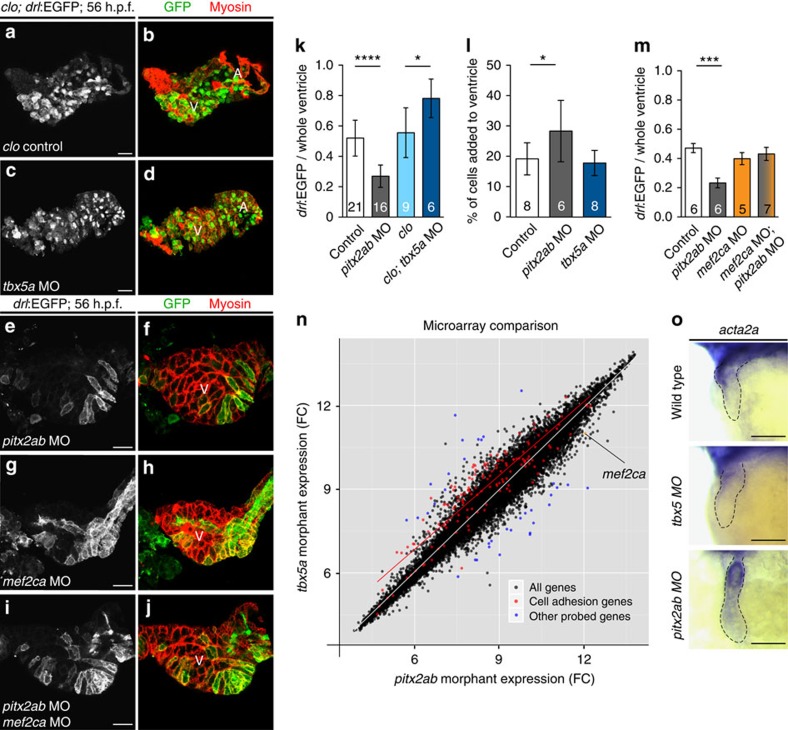
*tbx5a* and *pitx2ab* affect the relative contribution of FHF versus SHF to the heart tube. (**a**–**j**) Top-down 2-μm confocal sections through zebrafish 54–56 h.p.f. hearts; scale bar, 20 μm. (**a**–**d**) Hearts mutant for *cloche* (*clo*) to enable imaging of myocardium without endocardial and erythrocyte signal in *drl:EGFP* transgenics, stained for EGFP expression from *drl:EGFP* (green, monochrome channel image in right column) and myosin/MF20 (red); (**a**,**b**) *cloche*-only control, (**c**,**d**) morpholino knockdown of *tbx5a* leads to expansion of FHF. (**e**,**f**) Loss of *pitx2ab* results in diminishing of FHF and expansion of SHF. (**g**–**j**) Genetic interplay of *mef2ca* with *pitx2ab* on FHF versus SHF contribution in wild-type hearts. (**g**,**h**) Loss of *mef2ca* leads to only mild reduction of FHF contribution. (**i**,**j**) Concomitant loss of *mef2ca* and *pitx2ab* reverts the *pitx2ab* morpholino (MO)-mediated impact on FHF formation. (**k**) Graph displaying the ratio of FHF area labelled by *drl:EGFP* expression to the whole ventricular area determined by anti-myosin staining in hearts from experiments represented in **a**–**f**. (**l**) Graph showing percentage of newly added cardiomyocytes to the ventricle after photoconversion of *myl7:nlsKikGR* at 27 h.p.f. and imaged at 56 h.p.f. (see also Supplementary Fig. 6). (**m**) Graph showing the restoration of the FHF area labelled by *drl:EGFP* expression to the whole ventricular area determined by anti-myosin staining in hearts with combined loss of *pitx2ab* and *mef2ca* comparing with the single morphant or control hearts. Error bars in **k**–**m**=s.d., asterisks indicate significance, significance tested by two-tailed unpaired *t*-test, *P*<0.05. (**n**) At 54–56 hpf, whole-embryo microarray comparison of *tbx5a* versus *pitx2ab* knockdown reveals cell adhesion genes significantly deregulated (red dots); note the upregulation of the SHF regulator *mef2ca* on *pitx2ab* knockdown (orange dot). (**o**) mRNA *in situ* hybridization of *acta2a* as representative deregulated gene at 52–56 h.p.f.; closeups of heart regions, anterior to the left, in indicated conditions, dotted outlines indicate heart tube; scale bar, 100 μm (see also Supplementary Fig. 8 for more examples).

Heart tube elongation through the addition of SHF cardiomyocytes is hypothesized to represent an evolutionary step towards a multi-chambered heart. In amphibians and reptiles, a partial septum arises within a single ventricle, while in mammals the septum completely partitions the ventricle into left (FHF-derived) and right (SHF-derived) chambers^12^. A host of transcription factors influence cardiac septation, among which a steep gradient of Tbx5 expression between the left and right ventricle is proposed to drive this septation^12^, while Pitx2 directs remodelling of the SHF and chamber asymmetry^24^,^25^,^26^. Using *drl* expression to distinguish FHF from SHF descendants, we revisited *tbx5* and *pitx2* loss-of-function phenotypes and asked whether these factors, as proxy for septation regulators, influence the basic interactions of FHF and SHF in the two-chambered teleost heart. We employed morpholino-mediated gene knockdown of *tbx5a*^4^,^16^,^27^ and *pitx2ab* (Supplementary Fig. 5) in either *drl:EGFP* or *drl:EGFP*; *cloche* embryos to specifically monitor the developing myocardium (the *cloche* background removes any imaging bias through endocardium signal) (Fig. 4a–f); both genetic combinations gave identical results, consistent with our findings that both FHF and SHF myocardium are established despite the lack of endocardium in *cloche* (Fig. 3e,f). Knockdown of *tbx5a* caused an expanded FHF and restricted SHF territory, resulting in a predominantly FHF-derived heart (80% of ventricle versus 50% of ventricle in controls, *P*=0.0138, two-tailed unpaired *t*-test) (Fig. 4c,d,k). Conversely, *pitx2ab* knockdown had the opposite effect, with a markedly diminished FHF contribution (only 25% of ventricle, *P*<0.0001, two-tailed unpaired *t*-test) and a predominantly SHF-derived heart (Fig. 4e,f,k). Of note, *tbx5a* knockdown also decreased EGFP mean fluorescence levels, suggesting a potential regulatory input of *tbx5a* on the FHF-specific regulatory element in the *drl* reporter.

*tbx5a* and *pitx2ab* could influence the differential contribution of FHF and SHF by altering SHF cardiomyocyte migration to the heart tube or by specifying the corresponding ALPM cell fates before the bilateral heart field migration. To explore the rates of cell recruitment to the developing heart tube, we used targeted photoconversion of myocardial cells on *tbx5a* and *pitx2ab* perturbation in a myocardium-specific *myl7:nlsKikGR* transgenic line^9^. Using this readout, *tbx5a* knockdown modestly reduced the rate of cellular addition to the heart between 27 and 52 h.p.f., while *pitx2ab* impairment increased the rate of accrual of SHF cells into the heart in the same developmental time period (Fig. 4l; Supplementary Fig. 6). These data suggest that *tbx5a* and *pitx2ab* affect the effective contributions of FHF and SHF to the developing heart. In addition, both *tbx5a* and *pitx2ab* altered the morphology and cell size of ventricular cardiomyocytes, with *tbx5a* knockdown resulting in hearts with a morphology akin to linear heart tube stages^4^,^16^ (Supplementary Fig. 7). To resolve to what extent *tbx5a* and *pitx2ab* affect specification of FHF and SHF cell fates, we attempted to trace the *drl*-expressing cells on the same genetic manipulations in *drl:creERT2; ubi:loxP-EGFP-loxP-mCherry* transgenics; these efforts remained inconclusive, as we failed to observe significant *loxP* recombination following 4-OHT induction at 12 somite stage or later.

Microarray analysis of *tbx5a* and *pitx2ab* morphants at 52–56 h.p.f. catalogued complex effects on gene expression that parallel the effects on FHF and SHF in these experiments. Gene set enrichment analysis^28^ revealed a reciprocal effect on genes involved in ‘homophilic cell adhesion', with downregulation of these transcripts in *pitx2ab* morphants and upregulation in *tbx5a* morphants, as compared with wild-type embryos (q<0.005; Fig. 4n; Supplementary Fig. 8; Supplementary Table 1). Differentially regulated genes include cadherins, protocadherins, but also genes encoding cytoskeletal components, whose modulated expression following knockdown of either *pitx2ab* or *tbx5a* we confirmed using ISH analysis (Fig. 4o; Supplementary Fig. 8). The regulation of homophilic cell adhesion genes is a key feature during cardiomyocyte specification^29^, and our findings suggest the existence of discrete transcriptional cell adhesion programs downstream of *tbx5a* and *pitx2ab*.

Importantly, the transcription factor *mef2ca* previously implicated in SHF specification in mice^30^ and zebrafish^8^ was markedly upregulated in *pitx2ab* morphants (Fig. 4n). Consistent with previous findings^8^,^9^, reduction of *mef2ca* levels alone by morpholino knockdown modestly perturbed the ratio of FHF versus SHF based on *drl:EGFP* expression (Fig. 4g,h,m). Remarkably, in contrast to the increased SHF contribution defect observed on *pitx2ab* depletion, the concomitant loss of *pitx2ab* and *mef2ca* led to the proportionate reestablishment of SHF and FHF, albeit without restoring proper cell positioning (Fig. 4i,j,m) or physiologic integration (see below). Taken together, our data reveal reciprocal effects of the septation regulators *tbx5a* and *pitx2ab* on the relative contributions of FHF and SHF to cardiogenesis, and document a significant role for increased levels of *mef2ca* in the expansion of the SHF caused by *pitx2ab* loss of function.

### Intercellular coupling differences in FHF versus SHF

To evaluate the downstream impact of *tbx5a* and *pitx2ab* on FHF and SHF specification, we explored the physiology of these discrete compartments. The evolution from peristaltic to sequential contraction to support the complex vertebrate body plan is associated with the specification of physiologic electrical gradients regulated at least in part by pharyngeal Wnt11 in the zebrafish cardiac epithelium^31^. We reasoned that these gradients might result from fundamental functional differences between FHF and SHF cardiomyocytes, which our *drl:EGFP* reporter now allows us to locate.

**Figure 5:**
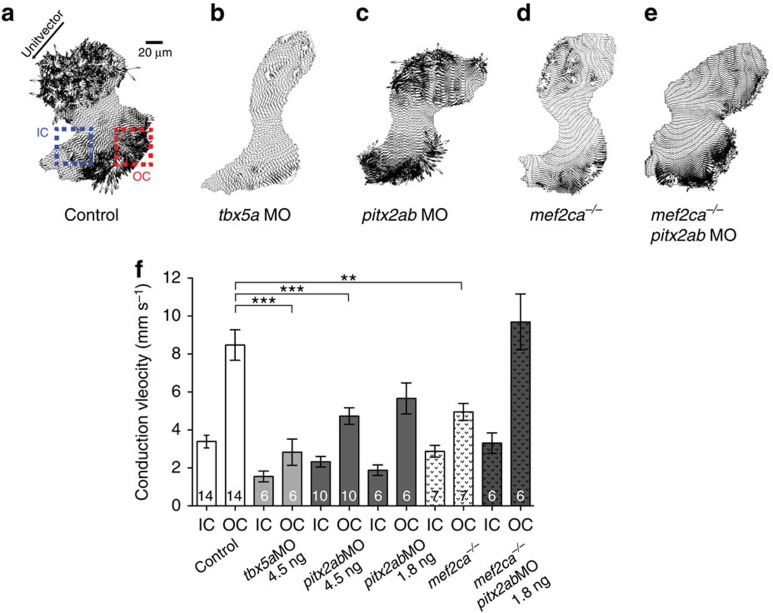
Myocardial conduction velocity gradient depends on proper integration of FHF with SHF. (**a**–**e**) Vector field maps of control (**a**) hearts revealing the difference in cardiomyocyte connectivity of outer curvature (OC, red square ROI), presumptive FHF and inner curvature (IC, blue square ROI), presumptive SHF. Hearts of *tbx5a* (**b**), *pitx2ab* (**c**) morphants and *mef2ca* mutants (**d**) show the marked slowing and change from complex to more uniform conduction in OC; partial recovery of these polarities occur in combined *mef2ca* mutant with loss of *pitx2ab* (**e**); unit vector shows the principal direction of propagation. (**f**) Mean estimated conduction velocities reveal marked deceleration of conduction velocities in *tbx5a*, *pitx2ab* morphant and *mef2ca* mutant hearts. Note the complete loss of the velocity coupling gradient in *tbx5a* morphant hearts and partial loss in *pitx2ab* morphant hearts and *mef2ca* mutants compared with control hearts. The reduction of *pitx2ab* in *mef2ca* mutants completely rescued the conduction velocity coupling gradient with marked acceleration of conduction velocities in OC; error bar=s.d., asterisks indicate significance, statistical significance tested with one-way analysis of variance, with Tukey's post test, *P*<0.05. All experiments performed at 54 h.p.f.

**Figure 6:**
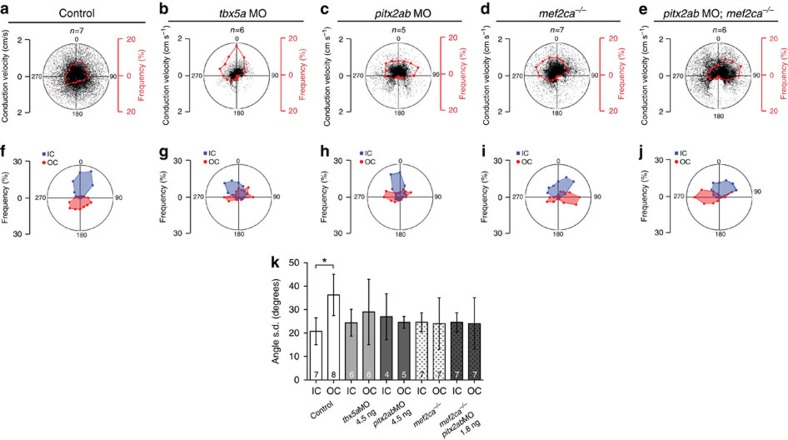
Myocardial connectivity depends on proper integration of FHF with SHF. (**a**–**e**) Vector polar plots, in which each point represents a vector of the vector field map plotted in respect to its magnitude (velocity) and direction (angle from unit vector), red points show the frequency of angle distribution at 22.5° intervals. (**a**) Wild type as control reference. Loss of *tbx5a* (**b**) results in almost uniform conduction, while loss of *pitx2ab* (**c**) and *mef2ca* (**d**) partially impairs the coupling gradients as compared to the control (**a**) hearts. Combined loss of *pitx2ab* and *mef2ca* (**e**) show marked recovery of large velocity vectors. Note the discrete absence of all angles between +160° and+200° in these hearts. (**f**–**j**) Frequency angle polar plots averaged from ROIs depicted in (Fig. 5a); majority of the vectors in control (**f**) hearts of OC/FHF point away from OFT revealing orthogonal mean coupling directions between OC/FHF and IC/SHF. Reduction of *tbx5a* (**g**) and *pitx2ab* (**h**) levels leads to complete loss of the orthogonal coupling and impairment in *mef2ca* mutants (**i**). Combined *mef2ca* mutants with loss of *pitx2ab* (**j**) partially rescues the orthogonal coupling direction. (**k**) Histogram of angle variability between inner and outer ventricular curvatures plotted as the angle s.d. in degrees. The angle variability is higher in outer curvature of control hearts, but this difference is diminished in the absence of *tbx5a*, *pitx2ab, mef2ca*, and combined *pitx2ab* morphant and *mef2ca* mutant hearts. Asterisks indicates significance, statistical significance tested with one-way analysis of varinace, with Tukey's post test, *P*<0.05. All experiments performed at 54 h.p.f.

In wild-type hearts, cardiomyocytes displayed consistent regional patterns of intercellular coupling with complex, strong and multidirectional isotropic cell–cell connections in the FHF-derived ventricular outer curvature where there is almost simultaneous activation of this entire region (Fig. 5a,f). In contrast, the SHF-derived inner curvature and distal outflow tract exhibit weak and linear anisotropic coupling patterns oriented along the main axis of depolarization of the heart (Fig. 5a,f). These findings were confirmed using quantitative polar plots of vector angle frequency across multiple hearts (Fig. 6a,f). The frequency angle polar plots as well as comparison of angle variability (Fig. 6k) not only revealed discrete intensity of electrical coupling between the *drl*-positive outer curvature and the *drl-*negative distal ventricle and outflow tract, but also identified almost orthogonal directions of mean connectivity in these two cellular compartments (blue and red regions of interests (ROIs) in Fig. 6f). Taken together, our observations reveal distinct coupling patterns in FHF- versus SHF-derived cardiomyocyte territories.

### Coupling depends on FHF versus SHF contribution

We next aimed to observe the impact of disrupting *tbx5a* (hearts with predominantly FHF-derived myocardium) or *pitx2ab* (hearts with predominantly SHF-derived myocardium) on the regional coupling patterns. Impaired *tbx5a* resulted in marked slowing of electrical conduction across the ventricle and the elimination of all heterogeneity of intercellular coupling, with the entire ventricle resembling the early heart tube before the addition of SHF (Figs 5b,f and 6b,g,k). *tbx5a* knockdown virtually eliminated all coupling complexity, with impulse propagation almost entirely restricted to directions along the main axis of the heart tube. We also observed marked loss of high-speed vectors with conservation of the low velocity vectors resulting in uniform slow, linear conduction typical of the more primitive peristaltic phase of cardiac development^31^.

*pitx2ab* knockdown also diminished conduction velocities in both the inner and outer curvatures of the ventricle (Fig. 5c,f), and appeared to reorganize the specific complex coupling patterns observed in the *drl*-positive regions of wild-type hearts (Fig. 6c,h,k). Although impaired *pitx2ab* had a more modest effect on the overall complexity of intercellular coupling, it eliminated the orthogonal outer curvature vectors required for sequential ventricular contraction (compare Fig. 6a,c, and red ROIs in Fig. 6f,h). Taken together, these data suggest that the development of physiologic patterns of cardiomyocyte connectivity requires a specific proportional interaction between FHF and SHF compartments, and that with perturbation of either compartment this nuanced patterning is lost.

To further relate our findings to our previous observations and to higher vertebrates, we tested the impact of the genetic interplay between *pitx2ab* and the SHF regulator *mef2ca*. Curiously, loss of *mef2ca*, which consistent with previous work^8^,^9^ and based on *drl:EGFP* reporter expression only mildly perturbs FHF versus SHF contribution (Fig. 4g,h), displayed similar physiological defects to those resulting from *pitx2ab* knockdown (Figs 5d,f and 6d,i,k). The joint loss of *pitx2ab* and *mef2ca* both rescued the overall velocity of impulse propagation and re-established the complex patterning of cardiomyocyte interaction observed in the normal ventricle, but the orthogonal vectors remained missing (Fig. 5e,f; Fig. 6e,j,k). This partial recovery reveals that not only is the correct ratio of FHF to SHF required, but also that there is a discrete requirement for additional directionally related coupling to achieve proper myocardial coupling.

## Discussion

The SHF is an ancient evolutionary entity of the vertebrate body plan^11^, yet its exact contribution to cardiac development, evolution and physiology remains vaguely understood. Here we provide data that propose a fundamental contribution of septation regulators to myocardium physiology before physical chamber delineation in the heart. A central finding is the modulation of FHF and SHF contributions by key transcription factors involved in cardiac septation in higher vertebrates when perturbed during development of the two-chambered heart in zebrafish. The resulting loss of myocardial coupling suggests a primordial contribution from the integration of the two heart fields to the establishment of efficient cardiac physiology, possibly through source sink mismatch.

The discovery of early *drl* expression in the entire lateral plate mesoderm identifies a molecular trait in the mesoderm margin that is common to the origin of all laterally developing tissues in the vertebrate embryo, including the FHF and SHF precursors. Unlike the restricted cardiovascular and hematopoietic expression of *FLK1* (refs 1, 32) or the broad mesodermal *Brachyury* activity^33^, the marginal *drl* domain specifically demarcates the lateral plate mesoderm and reveals an early, dedicated mesoderm subdivision before its expression becomes constrained to cardiovascular and haematopoietic lineages. Our lineage tracing results provide the first genetic confirmation for previous cell-based linage tracing efforts that fate mapped the lateral organ precursors^34^,^35^,^36^,^37^, and suggest the existence of an upstream regulatory program that demarcates the entire lateral plate mesoderm during gastrulation. Later *drl* reporter expression that confines in the myocardium to the FHF descendants seemingly diverges from endogenous detected mRNA expression that becomes undetectable after 24 h.p.f. (ref. 18) We hypothesize that, once taken out of genomic context, the isolated 6.35 kb regulatory element unmasks this previously unappreciated *drl* expression domain.

The structural and functional specialization of the four-chamber mammalian heart is deeply rooted in the discrete physiologic characteristics of FHF and SHF cardiomyocytes^12^. Beyond physical heart chamber separation, proportionate integration of distinctive cells from both FHF and SHF results in a polarized and tightly coupled myocardial syncytium that generates the sequential contraction of the ventricle, an indispensable step towards higher-pressure vertebrate circulation systems. Our work reveals that loss or excess of SHF cells results in the elimination of this balanced polarity and substantially reduces integration of cardiomyocytes across the entire ventricular myocardium. Our findings involving the downstream physiological impact of *pitx2ab* and *mef2ca* loss and their genetic interaction indicate that both the relative contribution of FHF versus SHF and specific integration of the SHF myocardium are required to establish subsequent heart muscle coupling. These findings suggest that additional, downstream coordination depends initially on SHF addition to the primary heart tube to properly pattern the myocardial syncytium. What pathways and which additional cardiac transcription factors mediate this downstream coupling, and what molecular differences between the FHF and SHF trigger this phenomenon, remains unknown. However, our data indicate that cell adhesion programs and their upstream regulatory pathways could mediate the proper coupling and topology of FHF and SHF. To what extent this process was retained in evolution towards terrestrial animals, particular in mammals, and what additional cardiac transcription factors fundamentally influence FHF versus SHF contribution to the heart warrants future experiments.

Altogether, our data suggest that the regional physiology within the un-septated teleost ventricle is under the control of the same basal transcriptional programs that support septation in higher vertebrates; consequently, these programs define potentially widely conserved functional myocardial gradients between FHF and SHF cell populations with distinctive physiological properties predicted to confer both mechanical advantage and electrical stability. Our findings propose that the separate systemic and pulmonary ventricles in higher animals derive from a functional partitioning that is already present in the more primitive teleost heart, where it conveys the selected physiological advantages that define the vertebrate circulation system.

## Methods

### Vectors and zebrafish strains

Zebrafish were maintained in accordance with Animal Research Guidelines at Brigham and Women's Hospital and Boston Children's Hospital. All PCR for cloning was performed using the Expand High Fidelity PCR kit (Roche). All subsequent MultiSite Gateway assemblies were carried out using LR Clonase II Plus (Life Technologies) according to the manufacturer's manual, if not stated otherwise. Tol2-mediated zebrafish transgenesis was performed by injecting 25 ng μl^−1^ transgene plasmid together with 25 ng μl^−1^ *Tol2* mRNA, followed by subsequent screening of positive *F0* founders and establishment of single-insertion transgenic strains through selection in subsequent generations. All experiments have been confirmed with at least two independent transgenic insertions.

The regulatory sequences of zebrafish *draculin* (*ZDB-GENE-991213-3)*^18^ sequence was PCR-amplified from BAC *DKEY-261J4* (*ZDB-BAC-050218-846*) with primers *5′-GTCAGCACCAGATGCCTGTGC-3′* (forward) and *5′-CCAAGTGTGAATTGGGATCG-3′* (reverse), and TOPO cloned into *pENTR5′* (Invitrogen) to create *pCM293*. *pCM293* includes a 6.347-kb genomic fragment *(−6.3drl*), including the non-coding exon 1, first intron, and non-coding sequences of exon 2 (see also Fig. 1a for details).

*Tg(−6.3drl:EGFP)* is a MultiSite Gateway assembly of *pCM293*, Tol2kit *#383* (*pME-EGFP*), *#302* (*p3E_SV40polyA*) and *#394* (*pDestTol2A2*) (in total *pDestTol2pA2_drl:EGFP*, referred to as *drl:EGFP*).

We created two independent transgene vectors for *drl:creERT2*: *Tg(−6.3drl:creERT2,cmlc2:EGFP)* and *Tg(−6.3drl:creERT2,alpha-crystallin:YFP)* derive from vectors *pDestTol2pA2_drl:creERT2*; *myl7-EGFP* (*pCM313*) and *pDestTol2CY_drl:creERT2*, *alpha-crystallin:YFP* (*pCM350*), respectively. *pCM283* was created by combining *pCM293*, *pENTR/D_creERT2* (ref. 38), and Tol2kit vectors *#302* and *#395* (*pDestTol2CG*); *pCM350* was created by combining *pCM293*, *pENTR/D_creERT2*, Tol2kit vector *#302* and *pDestTol2CY* (*pCM326*, derived from inserting a *Asp718I*-flanked PCR product of *alpha-crystallin:YFP* (*CY*)^39^ into the *Asp718I* site in Tol2kit *#394 i*n the same orientation as the Multisite Gateway cassette). Detailed digital plasmid maps of these vectors are available on request.

Additional transgenic and mutant lines used in this study included *lmo2:loxP-dsRED-loxP-EGFP* (used both as red fluorescent *lmo2* reporter and *loxP* switch)^40^, *ltbp3:TagRFPA2:cre* and *myl7:loxP-AmCyan-loxP-ZsYellow*^7^*, FlEx*^41^, *ubi:loxP-GFP-loxP-mCherry* (*ubi:Switch*)^38^, *cdh17:loxP-EGFP-loxP-mCherry*^42^, *cloche*^*M39*^ (ref. 23), *myl7:nlsKikGR*^9^, and *mef2ca*^*b1086*^ (ref. 43).

CreERT2/*lox* experiments were performed by crossing male *creERT2* driver transgenics with female *lox* reporter carriers, and the embryos were induced with a final concentration of 10 μM 4-OH-tamoxifen (4-OHT, Sigma H7904)^38^,^44^ in E3 embryo medium (and later washed out) at the time points indicated in the text.

Microscopy was performed on a Zeiss 710 NLO confocal microscope (whole-mount samples), Leica SP5 or SP8 (fixed samples) a Zeiss SteREO Discovery V8 with AxioCam HRc camera for stereo microscopy, and a Zeiss Axioskop 2 plus with Hamamatsu ORCA-ER camera for upright microscopy.

### Zebrafish transgenics and morpholinos

The *tbx5a* morpholino sequence was adapted as previously reported (*MO2-tbx5a*)^16^ and has the sequence *5′- CCTGTACGATGTCTACCGTGAGGC-3*′. Our *pitx2ab* ATG morpholino with sequence *5′-TGGGAGTCCATTTAGTAGGTTATAT-3′* was validated to cause left–right asymmetry defects, including cardiac phenotypes (Supplementary Fig. 5).

### Cell sorting and microarray analysis

For *drl:EGFP* arrays, three independent times the same family of homozygous *drl:EGFP* transgenic zebrafish was mass-mated and their embryos were collected at 2 somite stage. The embryos were de-chorionated, homogenized in PBS with 5% FCS with a hand-held motor pistil, and filtered twice through 40-μm cell strainers (BD Biosciences). Propidium iodide was added and the cell suspension sorted using a BD LSR II Flow Cytometer (BD Biosciences) for EGFP positive, propidium iodide negative, and EGFP negative, propidium iodide negative into Trizol LS (Life Technologies) with subsequent mRNA isolation as per the manufacturer's protocol and added phenol/chloroform precipitation. The samples were then processed by the Boston Children's Hospital Molecular Genetics Core Facility for hybridization and analysis using Affymetrix microarray chips based on zebrafish genome annotation Zv9.

Normalized (MAS background correction and RMA normalization) gene-level expression data were computed from Affymetrix ZebGene-1_1-st CEL files using the Affymetrix ExpressionConsole software. Regulated gene lists were computed from the normalized data using the R/Bioconductor package ‘siggenes' and an false discovery rate threshold of 0.09.

To validate candidate gene expression patterns, total RNA was isolated from bud-24 h.p.f. embryo mix using Trizol LS extraction (Invitrogen), followed by reverse transcription using the SuperScript III cDNA kit (Invitrogen) to create a complementary DNA pool. One microlitre of complementary DNA was used in 50 μl PCR reactions using the Expand High Fidelity PCR kit (Roche) and gene-specific forward plus reverse primers, which contained 5′ T7 consensus sequences. The resulting PCR products were checked for size, purified using the QiaQuick PCR Purification Kit (Qiagen) and transcribed into Digoxigenin (DIG)-labelled ISH probes (Roche); primer sequences for individual tested candidate genes are available on request.

### *tbx5a* versus *pitx2ab* microarray and analysis

Samples for *tbx5a* versus *pitx2ab* morpholino comparison were collected from three independent experiments of morpholino-injected siblings and uninjected wild-type controls at 56 h.p.f. by morphology. RNA was isolated using Trizol LS (Life Technologies) as per the manufacturer's protocol. The samples were processed by the Boston Children's Hospital Molecular Genetics Core Facility for hybridization and analysis using Affymetrix Zv9 microarray chips.

Microarray data were normalized and processed using the Oligo package for the R statistical framework. Following normalization, values for replicates were averaged and fold change over control was calculated for each gene in both *pitx2ab* and *tbx5a* morphants. For gene set enrichment analysis, genes were pre-ranked based on fold change in either *pitx2ab* and *tbx5a* morphants, and examined for enrichment of gene sets that included predicted gene phenotypes (only gene phentoypes predicted above an 80% precision level), GO terms, characterized gene lists from MSigDB and human tissue-specific expression. For MSigDB and human tissue expression analysis, the pre-ranked list only included genes with identified one-to-one human orthologs (Ensemble). To generate the human tissue expression gene sets, human tissue-specific transcript expression data were obtained through the gene expression omnibus and mapped through Affymetrix expression tags to human genes. Average transcript expression of higher than 100 was taken as indicating expression of a given gene within a given tissue. Only those gene sets with q<0.05 were reported. Expression patterns of candidate genes were performed as outlined above for the microarray validation on sorted *drl:EGFP* cells.

### Fluorescence measurements and propagation velocity calculation

The measurement of action potentials and the estimation of conduction velocities from isolated zebrafish embryo hearts were performed as we previously described^31^,^45^. In detail, for the measurements of action potentials, hearts isolated from wild-type zebrafish embryos or morpholino-injected embryos at 54 h.p.f. were stained with the transmembrane potential-sensitive dye di-8-ANEPPS (Invitrogen). Fluorescence intensities were recorded using a high-speed CCD camera (CardioCCD-SMQ, Redshirt Imaging) at 2,000 frames per second. Local activation times were defined as the times at which action potentials reached 50% of their amplitude during the depolarization phase. This criterion for defining electrical activation was found in computer simulations to correlate well with the time at which the maximum depolarizing Na+ current occurs during propagation^46^. Local conduction velocity vectors were estimated by fitting a second-order polynomial surface to the activation times using an established algorithm^47^. Conduction velocities and angles were averaged in ROIs, 16 × 16 pixels in size, covering an area of about 35 × 35 μm^2^ within the outer curvatures and inner curvatures of the cardiac chambers.

### Angle vector map analysis

To evaluate local directions of depolarization, the angles of the conduction velocity vectors were calculated with respect to a unit vector that was determined by the geometry of each heart. It was defined as a vector pointing from the inner curvature of the atrium, that is, the region that in most hearts activated first, towards the outflow tract, which activated last. Thus, the unit vector could be described as the principal direction of propagation. All angles that were observed in the outer curvatures or inner curvatures of all hearts for a given genotype were divided into 22.5° wide-angle intervals (ranging from 0° to 360°) and presented as frequency polar plots. The radial coordinate was defined (in %) as the number of velocity vectors that were found within a given frequency interval divided by the total number of vectors.

### Immunostaining, confocal microscopy, and image analysis

Hearts, isolated from 26, 52–56 and 72 h.p.f. zebrafish embryos, were fixed in Prefer fixative (Anatech Ltd), stained with primary antibodies: rabbit anti-GFP (G1544, Sigma) 1:100; mouse anti-myosin (MF20; Developmental Studies Hybridoma Bank) 1:50; mouse zn-8 (Developmental Studies Hybridoma Bank) 1:50; and secondary antibodies: donkey anti-rabbit or mouse Alexa-Fluor-488 or -555 conjugated (Invitrogen) 1:1,000 and mounted in the ProLong Gold antifade reagent with 4,6-diamidino-2-phenylindole (Invitrogen). Confocal images were obtained using Leica SP5, SP8 and Nikon C1, and processed using ImageJ/Fiji, Packing analyser v2.0, Paint Shop Pro, Photoshop, and CombineZP. Graphs for statistical analysis were generated in GraphPad Prism 5.

## Additional information

**Accession codes**: Microarray data have been deposited in the GEO database under accession codes GSE70881 and GSE70750.

**How to cite this article:** Mosimann, C. *et al.* Chamber identity programs drive early functional partitioning of the heart. *Nat. Commun.* 6:8146 doi: 10.1038/ncomms9146 (2015).

## Supplementary Material

Supplementary Information Supplementary Figures 1-8 and Supplementary Table 1
