# Supplementary material for: Chamber identity programs drive early functional partitioning of the heart
Source: Nat Commun. 2015 Aug 26;6:8146. doi: 10.1038/ncomms9146 (PMC4560818; doi:10.1038/ncomms9146)
Supplement: Supplementary Information — Supplementary Figures 1-8 and Supplementary Table 1 [file ncomms9146-s1.pdf]

Supplementary Figure 1 - Mosimann, Panáková, et al.

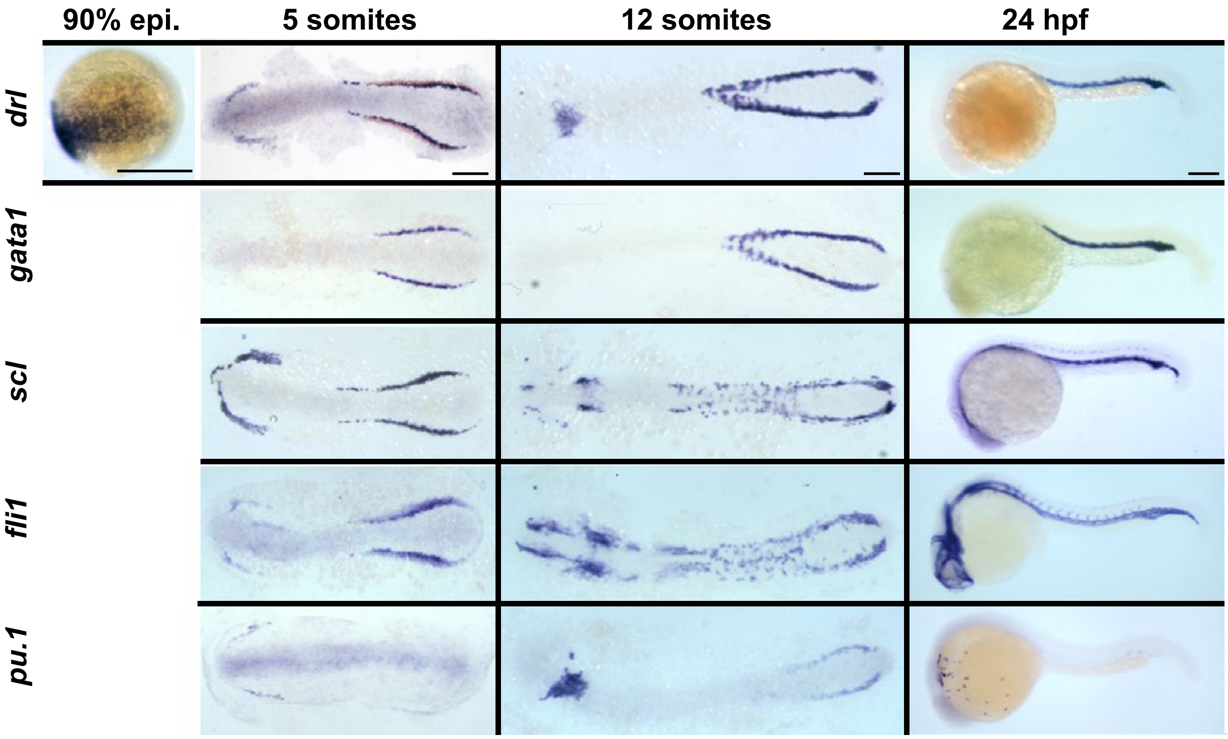

Supplementary Fig. 1. Zebrafish *drl* is transcribed earlier than traditional blood markers, labels the forming lateral mesoderm, and subsequently restricts expression into cardiovascular and hematopoietic lineages. Panel of classic marker genes expressed in hematopoietic populations, scale bars = 200 µm; the epiboly-stage embryo is animal to the top, ventral (V) to the right, dorsal (D) to the left; later stages (flat mounts and 24 hpf whole mounts) are anterior (A) to the left, posterior (P) to the right; note prominent expression of all genes in the Intermediate Cell Mass (ICM) at 24 hpf, except the myeloid marker *pu.1* which is expressed in the anterior myeloid precursors.

Supplementary Figure 2 - Mosimann, Panáková, et al.

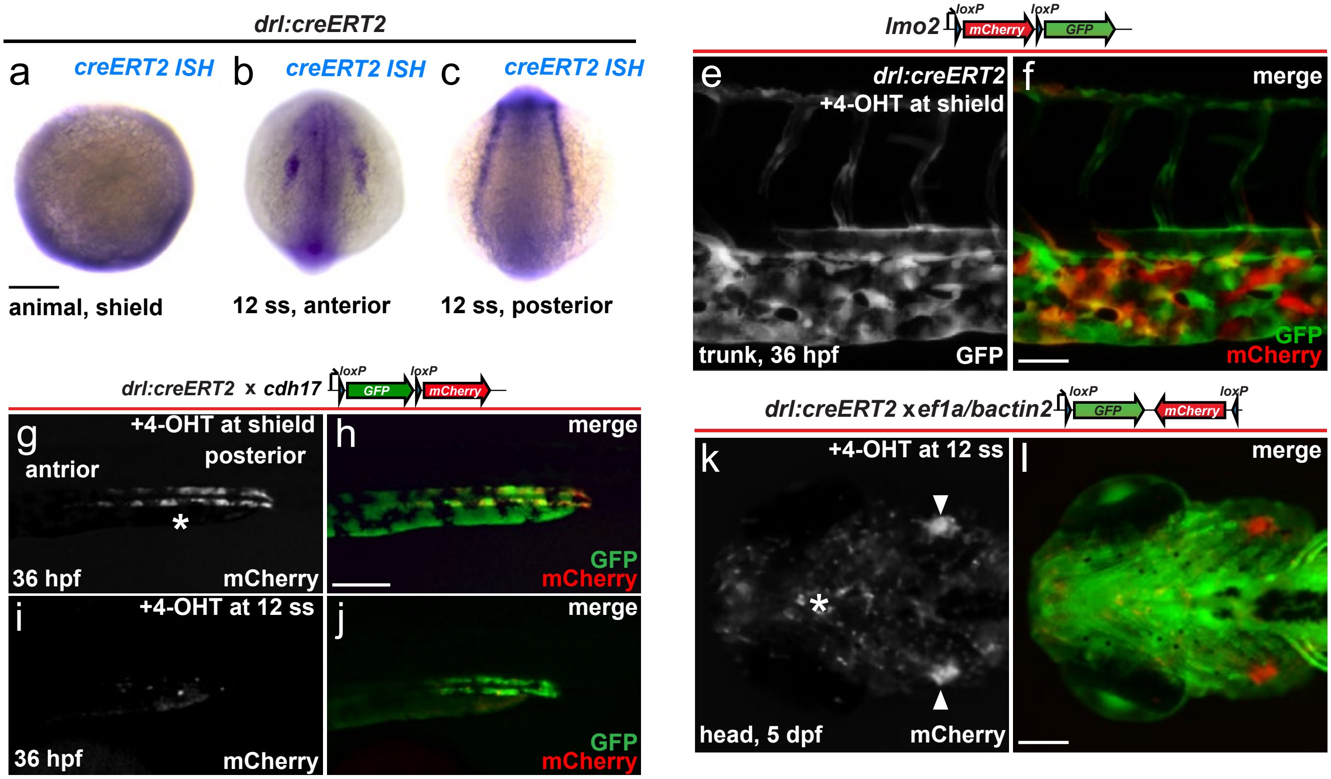

Supplementary Fig. 2. *drl* reporter expression refines to anterior and posterior cardiovascular lineages over time. (**a-c**) mRNA *in situ* hybridization (ISH) for *creERT2* in *drl:creERT2* embryos shows expression at shield stage (**a**, animal pole view, ventral on top) at the embryo margin, and at 12 somite stage (**b**, **c**) shows the separation of *drl* reporter expression into anterior and posterior lateral mesoderm; scale bar = 100 µm (**e, f**) Vascular lineage tracing confirmed using *drl:creERT2* crossed to the endothelial-specific *loxP* reporter *lmo2:Switch* (schematic) and 4-OHT-induced at shield stage, tracing (GFP signal, green) shown in the trunk at 36 hpf; scale bar = 50 µm. (**g-j**) Transgenic zebrafish carrying *drl:creERT2* crossed to *cdh17:loxP-GFP-loxP-mCherry* (schematic) were induced with 4-OHT at shield or 12 somite stage; mCherry expression in the renal tubules reveals *drl* expression in their precursors during gastrulation (**g**, **h**), but not at later stages of somitogenesis (**i**, **j**); scale bar = 100 µm. (**k**, **l**) Definitive hematopoietic lineages descend from *drl*-expressing cells at 12 ss, as shown in a 5-day larva with tracing to the developing bilateral thymus (arrowheads, **k**) and tissue-infiltrating leukocytes (asterisks, **k**) using the *ef1a/beta-actin2*-based *FlEx* reporter line (schematic); scale bar = 100 µm.

Supplementary Figure 3 - Mosimann, Panáková, et al.

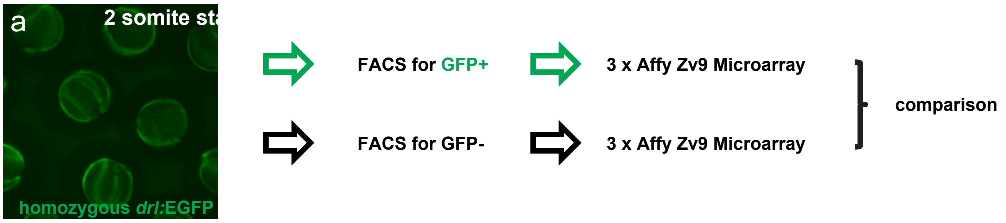

b

8-fold and more enriched expression in *drl:EGFP* territory

R.fold	Gene	lateral expression
24.1	*PDE4B*	n/d
20.0	*tmem88a*	x
17.0	*tbx20*	x
15.7	*etv2*	x
14.5	*scl/tal1*	x
13.5	*gata5*	x
13.5	*wu:fb37b09*	n/d
13.3	*gata6*	x
13.2	*osr1*	x
12.9	*hand2*	x
12.4	*drl*	x
11.9	*lgals3l*	x
11.2	*fgfrl1b*	x
10.9	*sox32*	forerunner cells
10.8	*hlxb9lb*	x
10.6	*si:dkey-261j4.3*	n/d
10.4	*pcdh12*	x
10.0	*hbbe1*	x
10.0	*hbae1*	x
9.8	*tbx1*	x
9.7	*ccdc85ca*	n/d
9.7	*fam176a*	x
9.6	*sox17*	forerunner cells
9.5	*pcdh10a*	x
9.4	*hnf1bb*	x
9.3	*pltp*	x
9.1	*nrp1b*	x
9.0	*si:ch211-163l21.8*	n/d
8.7	*rbpms2*	x
8.7	*setd3*	n/d
8.6	*si:ch73-234b20.5*	x
8.5	*CU468041.1*	x
8.3	*lmo2*	x
8.2	*si:dkey-95h12.1*	n/d
8.2	*gfra1a*	x
8.1	*gpm6ab*	n/d
8.1	*prrx1a*	x

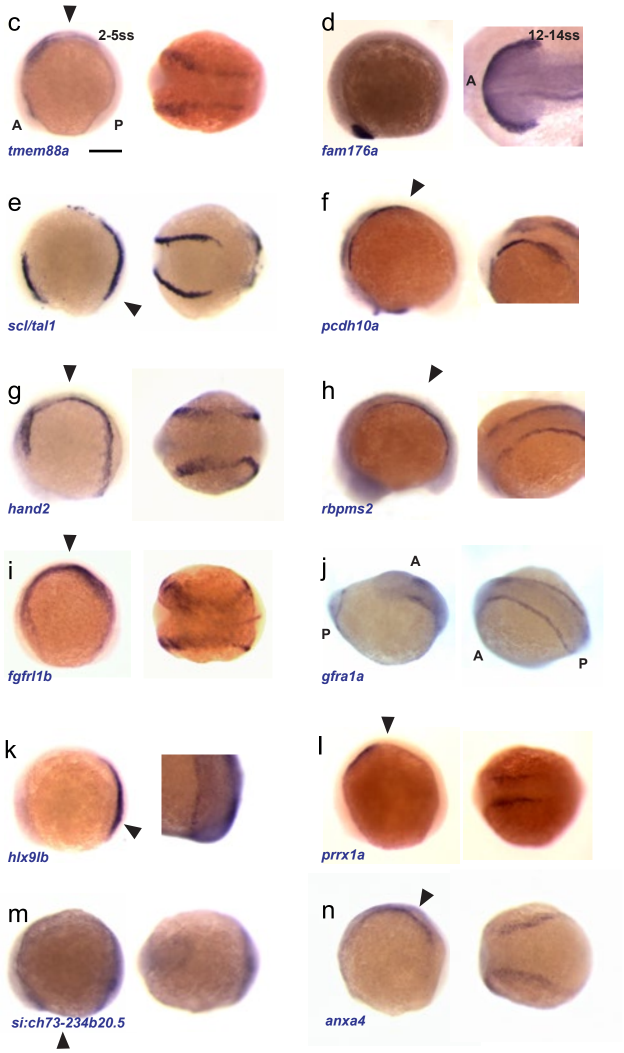

Supplementary Fig. 3. The transcriptome of the *drl*-positive lateral mesoderm at the beginning of segmentation. (**a**), schematic of the performed Affymetrix Zv9 microarray experiment; 2 somite stage *drl:EGFP* homozygous embryos were collected, dissociated, their EGFP-positive and –negative cell populations separated by FACS, the isolated RNA from both populations was subjected to microarray analysis, and the resulting gene expression lists from both populations compared. This resulted in a list of genes that are preferentially expressed in the *drl*-positive lateral territory. (**b**), excerpt of the enriched gene list (8-fold or higher), including expression analysis by RNA ISH. (**c-n**), validation of lateral expression by mRNA ISH for selected candidates from the microarray analysis; for each gene, a lateral view (left, anterior (A) to the left, posterior (P) to the right) and detail view (right, point of view shown by arrow head in lateral view) is shown for 2-5 ss embryos if not depicted otherwise; scale bar = 100 µm.

Supplementary Figure 4 - Mosimann, Panáková, et al.

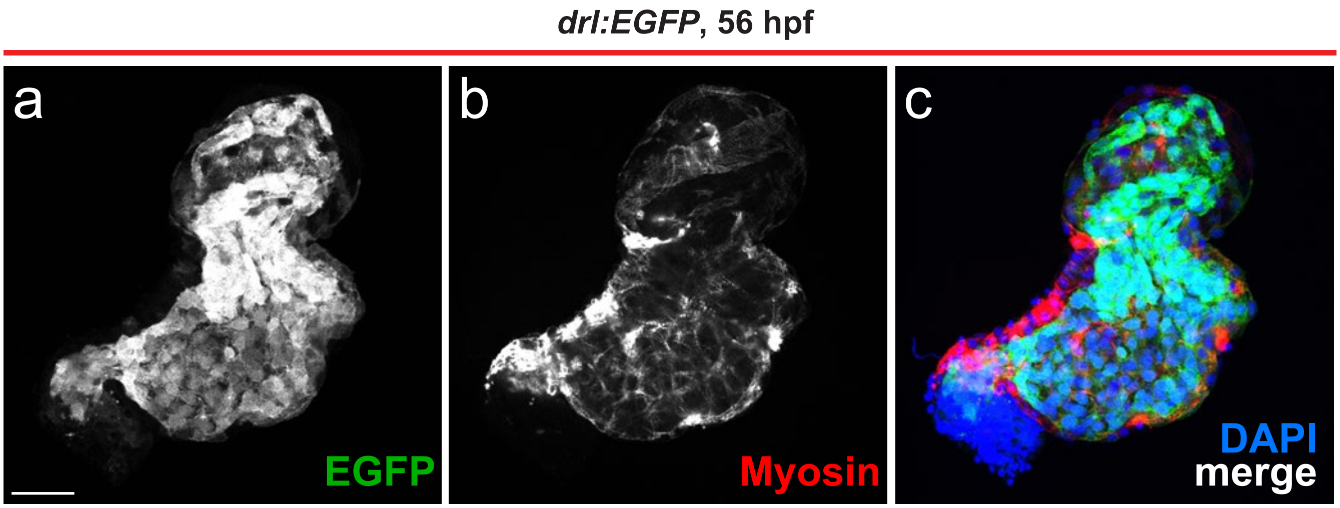

Supplementary Fig. 4. Endocardial activity of *drl*-expressing reporters. (**a**-**c**) Medial confocal section through a 56 hpf zebrafish heart transgenic for *drl:EGFP* (green) to reveal expression of *drl:EGFP* in the central Myosin-negative endocardium; counter-stained cardiac Myosin/MF20 (red), DAPI (blue) as nuclear stain. Ventricular outer curvature (OC) myocardium is clearly GFP-positive, while GFP signal is absent from inner curvature (IC) myocardium. Scale bars = 50 µm.

Supplementary Figure 5 - Mosimann, Panáková, et al.

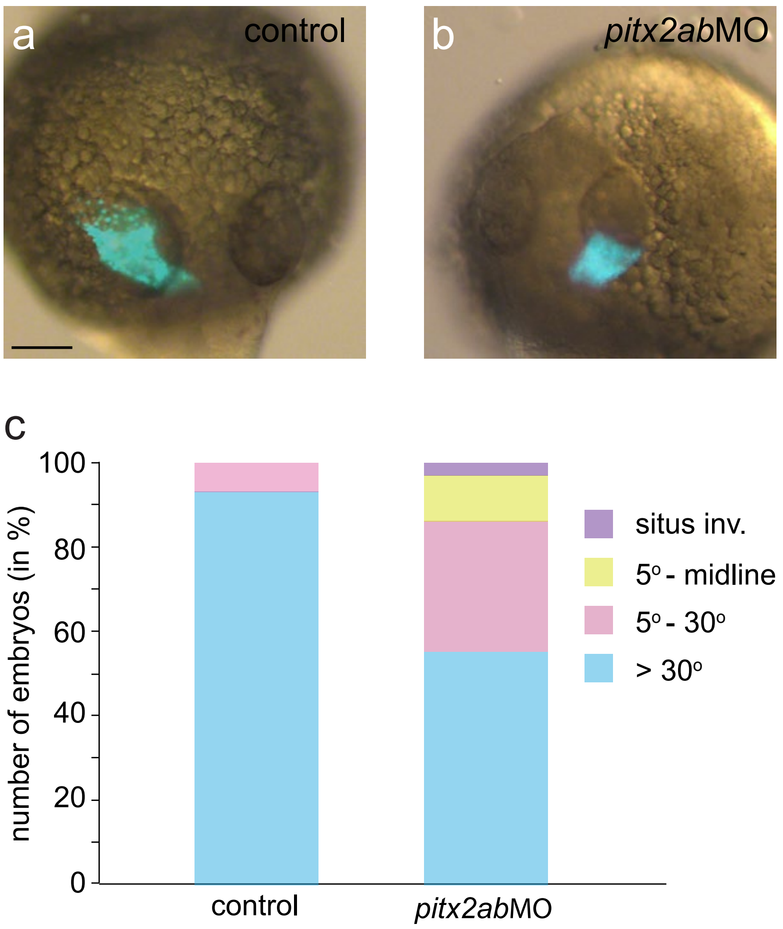

Supplementary Fig. 5. Validation of morpholino-mediated *pitx2ab* knockdown. Scale bar = 100 µm. (**a-c**) 24 hpf heart tube positioning in wildtype control (**a**) shows normal left tilt of the heart tube referred to as jog, while *pitx2ab* morphants produce random jog (**b** shown to the right, **c** statistical representation of phenotype distribution).

Supplementary Figure 6 - Mosimann, Panáková, et al.

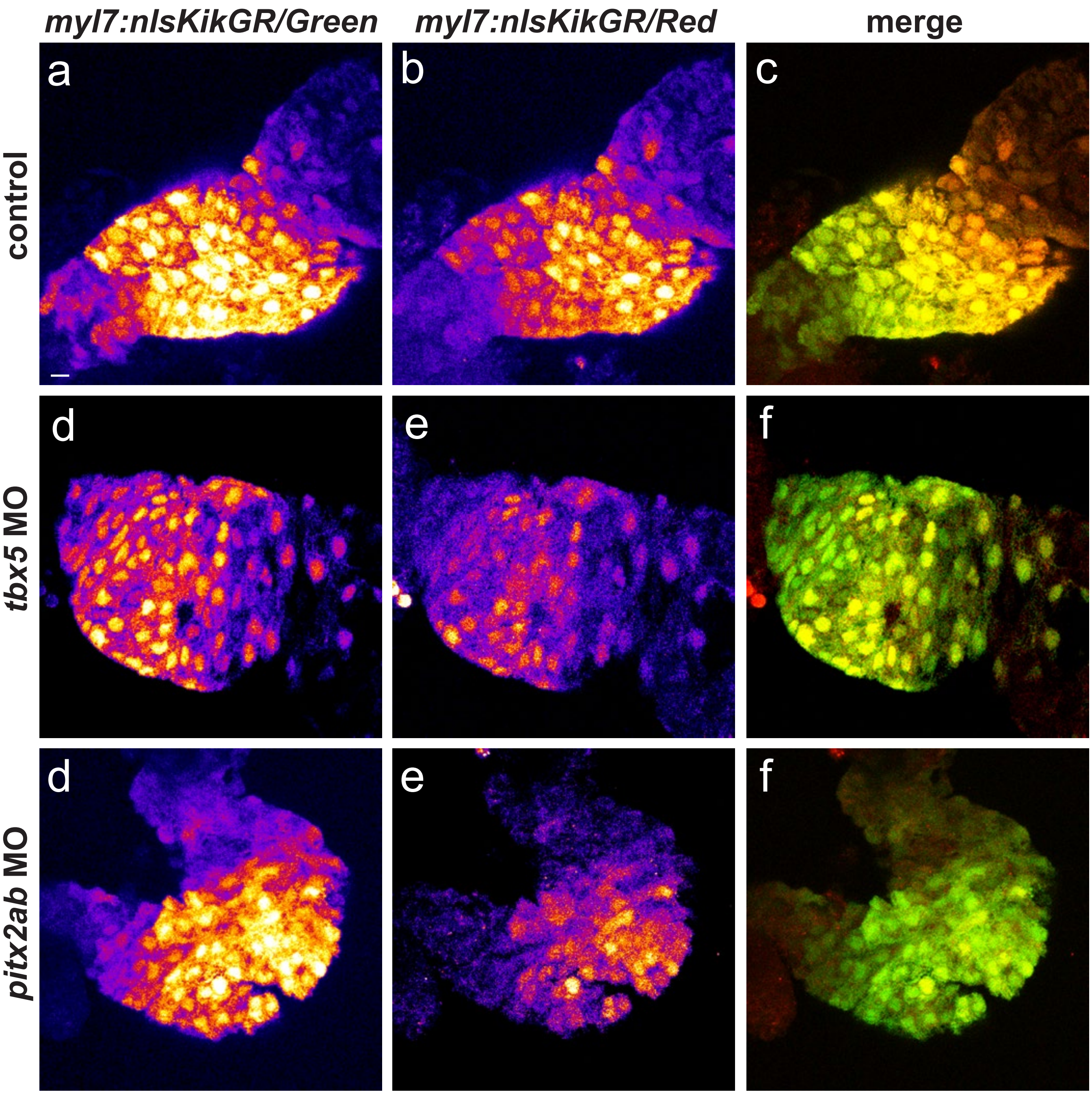

Supplementary Fig. 6. Impact of *tbx5a* and *pitx2ab* knockdown on SHF addition to the FHF. Scale bar = 10 µm. (**a-i**) Photoconversion-mediated lineage tracing of the SHF cardiomyocytes addition to the linear heart tube. Photoconversion of cardiomyocyte-specific Tg(*myl7:nlsKikGR*) was performed at 365 nm for 2 minutes at 27 hpf and the hearts imaged at 56 hpf; the total conversion to RFP was visually confirmed. **a**, **d**, **g**, shows the green channel, **b**, **e**, **h**, shows the red channel, merge is shown in **c**, **f**, **i**. All GFP-positive nuclei are the newly added cardiomyocytes after 27hpf, RFP-labeled nuclei represents all cardiomyocytes of the linear heart tube prior to photoconversion, yellow in merge (**c**, **f**, **i**). **a-c,** control hearts, **d-f**, hearts from *tbx5a* morphants show mild decrease in newly added cardiomyocytes, **g-i**, hearts from *pitx2ab* morphants display increase in cardiomyocyte recruitment.

Supplementary Figure 7 - Mosimann, Panáková, et al.

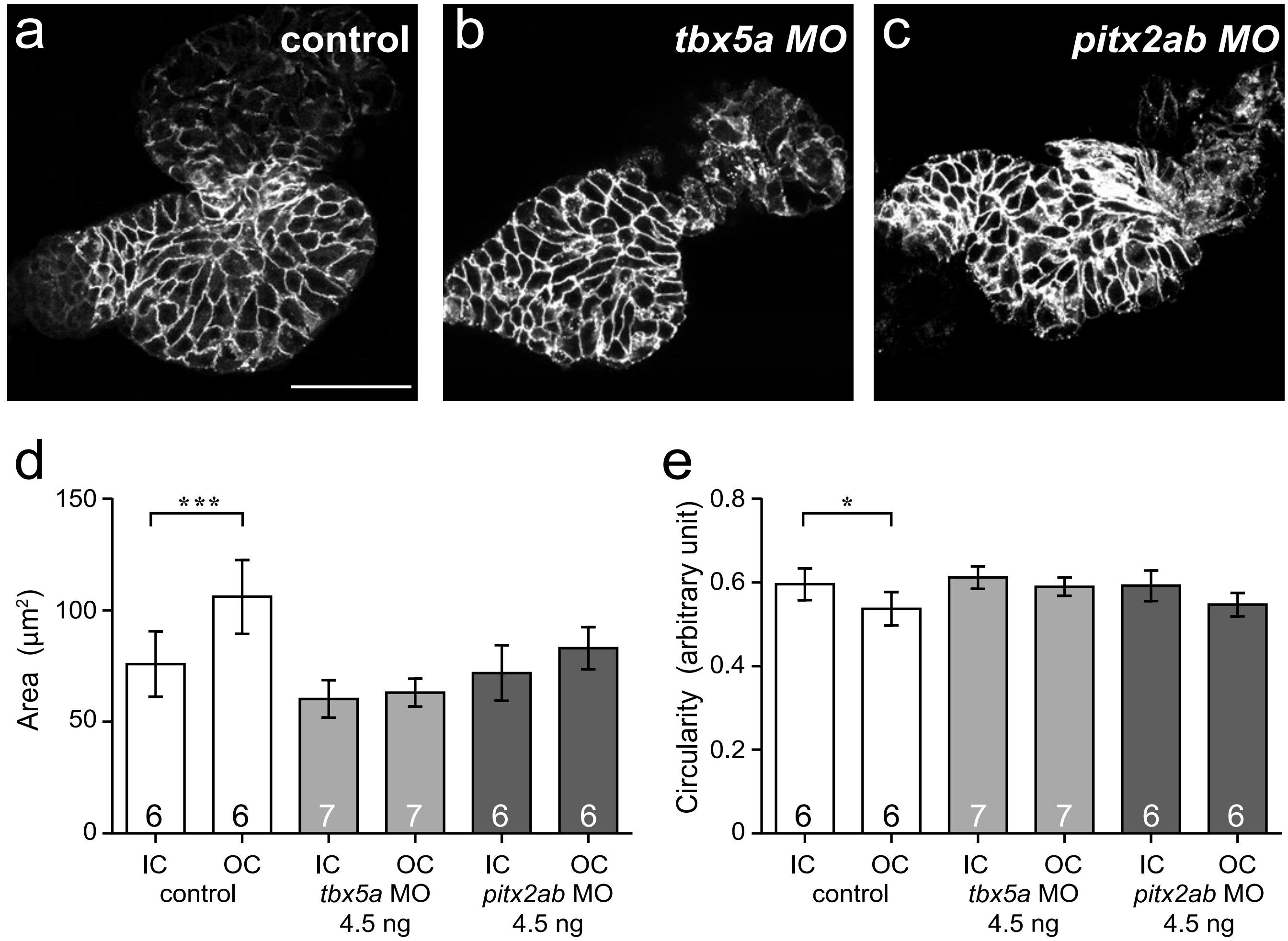

Supplementary Fig. 7. Morphology of hearts in loss of *tbx5a* and *pitx2ab* is altered. (**a-c**) Apical 1 μm confocal sections through 72 hpf zebrafish hearts stained for membrane marker (zn-8) from control (**a**), *tbx5a* morpholino knockdown (**b**), and *pitx2ab* morpholino knockdown (**c**) embryos; scale bar = 50 μm. (**d**, **e**) Graphs compare the area (**d**) and circularity (**e**) of the ventricular cardiomyocytes between inner and outer curvature in control hearts and in heart with the loss of *tbx5a* and *pitx2ab*. Statistical significance tested with One-way Anova, with Tukey's post test, $p<0.05$. Both, *tbx5a* and *pitx2ab* knockdown results in smaller (**d**) and more circular (**e**) cells. The changes are more apparent in the hearts from *tbx5a* knockdown embryos resembling the morphology of the primitive linear heart tube stage.

Supplementary Figure 8 - Mosimann, Panáková, et al.

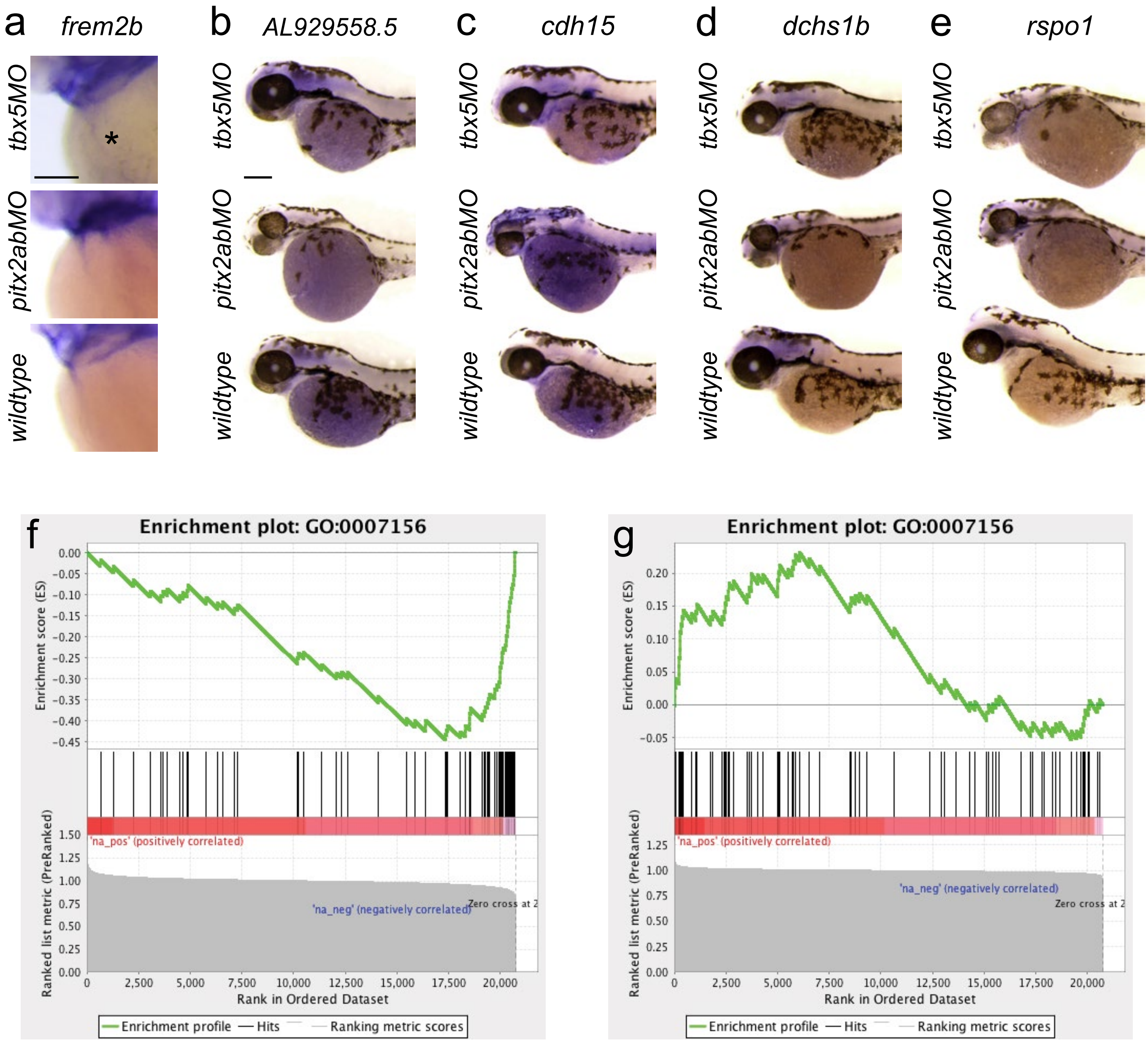

Supplementary Fig. 8. Microarray candidates and GSEA from *tbx5a* versus *pitx2ab* morphant analysis. (**a-e**) mRNA *in situ* hybridization of genes that are significantly de-regulated in *tbx5a* versus *pitx2ab* knockdown conditions; images show head portions of zebrafish embryos at 52-56hpf, anterior to the left; scale bars = 100 µm. (**f**, **g**) Analysis of transcriptional profiling results; Gene Set Enrichment Analysis (GSEA) was run using gene lists pre-ranked based on transcriptional changes following knockdown of either *pitx2ab* or *tbx5a* (see **Methods**). Genes annotated as being involved in 'homophilic cell adhesion' (GO:0007156) were found to be significantly down-regulated in *pitx2ab* (**f**) morphants, and up-regulated in *tbx5a* (**g**) morphants ($q < 0.05$). Within each panel, the enrichment score (ES; upper plot) is a running sum statistic, wherein positive deviation from 0 indicates enrichment for a given gene set at the top of the ranked list, and negative deviation indicates enrichment at the bottom. Barcoding (middle plot) indicates observation of genes from the tested gene set at the indicated position of the ranked list. The bottom plot shows the value of the ranking metric throughout the ordered set.

Supplementary Table 1 - Mosimann, Panáková, et al.

EnsID	Gene Name	fc pitx2	fc tbx5
ENSDARG00000087940	BX323820.1	1.24968054	1.004838129
ENSDARG00000069473	frem1a	1.07649046	0.976489157
ENSDARG00000019726	celsr2l	1.05804161	0.974822079
ENSDARG00000074369	frem2b	1.04309679	0.984909698
ENSDARG00000045403	CABZ01090951.1	1.03824797	0.990486055
ENSDARG00000024371	cdh1	1.03469349	1.01309126
ENSDARG00000092450	si:dkey-173l11.3	1.03264144	0.999870437
ENSDARG00000018923	FAT2	1.0326209	0.987662207
ENSDARG00000052232	pcdh15a	1.03091294	1.00313398
ENSDARG00000087126	FP245455.1	1.03060741	1.02457116
ENSDARG00000017591	fat1	1.03035885	0.980478918
ENSDARG00000062608	pcdh1g2	1.02876044	1.042587419
ENSDARG00000058259	celsr1b	1.0253861	0.972178112
ENSDARG00000008127	pcdh15b	1.02445006	1.013102365
ENSDARG00000095430	zgc:123181	1.02357711	1.036662384
ENSDARG00000057773	si:dkey-27i16.3	1.02338216	0.995123222
ENSDARG00000068191	cdh15	1.01915002	0.989320553
ENSDARG00000058546	si:dkey-30c15.12	1.01708046	0.994055119
ENSDARG00000035796	si:ch211-206i14.2	1.01701507	1.007563512
ENSDARG00000005112	cdh17	1.01597583	0.996452008
ENSDARG00000075960	CDH24 (1 of 2)	1.01360808	1.009821275
ENSDARG00000078404	CDH26	1.01310635	0.985132569
ENSDARG00000077570	CDH16	1.01136868	1.003446462
ENSDARG00000095360	si:ch211-213b8.4	1.00937371	1.02616892
ENSDARG00000070025	DCHS1 (1 of 2)	1.00628395	0.986217408
ENSDARG00000017649	CDH7 (1 of 2)	1.00582169	0.995529588
ENSDARG00000060830	FAM83H (2 of 2)	1.00509293	0.990597511
ENSDARG00000069185	celsr1a	1.00288413	0.990009466
ENSDARG00000075549	cdh5	1.00275658	1.008299739
ENSDARG00000044048	prnpb	1.00195859	0.953012547
ENSDARG00000089176	CDHR2 (2 of 2)	1.00118411	1.005467932
ENSDARG00000004643	cdhr1a	0.9991	1.008732392
ENSDARG00000007561	cdh23	0.99683352	1.009890099
ENSDARG00000021442	cdh11	0.9961231	0.992573861
ENSDARG00000014522	cdh6	0.99491589	0.983112278
ENSDARG00000018693	cdh2	0.99218507	0.979475461
ENSDARG00000074991	CDH19	0.99165734	0.985443487
ENSDARG00000035660	si:ch211-193k19.1	0.98892123	1.002311832
ENSDARG00000090275	CDHR5 (2 of 2)	0.98517063	0.991858159

ENSDARG00000079247	si:dkey-22o22.2	0.9847161	0.997820168
ENSDARG00000055305	ret	0.98215505	0.97322505
ENSDARG00000023542	cdh7	0.98003261	0.989719557
ENSDARG00000045665	FAT4	0.97794861	0.989708405
ENSDARG00000060610	pcdh7b	0.97765589	1.01698595
ENSDARG00000075268	CABZ01034923.1	0.97753773	1.023656889
ENSDARG00000034344	pcdh19	0.9765819	0.993951373
ENSDARG00000052494	pcdh18b	0.9763079	0.982816677
ENSDARG00000076594	PCDH12	0.97563846	0.998822318
ENSDARG00000078088	CR339051.1	0.97544847	1.007322077
ENSDARG00000033840	FAT3	0.97534296	0.998267174
ENSDARG00000071894	pcdh2g3	0.96917781	0.9815546
ENSDARG00000036175	pcdh1b	0.96908761	1.003026252
ENSDARG00000060637	clstn2	0.96832727	0.992002415
ENSDARG00000031720	clstn1	0.96789779	0.979453389
ENSDARG00000060638	CLSTN2 (1 of 2)	0.96314352	1.011384676
ENSDARG00000036424	PCDH20	0.96182338	1.008769206
ENSDARG00000054274	pcdh10a	0.96164774	0.990581773
ENSDARG00000078190	zgc:193751	0.95889253	1.005726256
ENSDARG00000079850	dchs1b	0.9587979	0.967569051
ENSDARG00000075724	pcdh2g1	0.95821391	1.008117677
ENSDARG00000055825	celsr3	0.95738893	0.981420691
ENSDARG00000074386	CDH12 (1 of 2)	0.95736595	0.998751855
ENSDARG00000077771	pcdh2g28	0.9560345	1.025879077
ENSDARG00000074595	pcdh2aa3	0.95602607	1.059356695
ENSDARG00000089805	pcdh18a	0.95598575	0.981046223
ENSDARG00000071893	pcdh2g4	0.95567123	1.078366477
ENSDARG00000074959	pcdh2g3	0.95554817	1.042684765
ENSDARG00000019063	FAT1 (1 of 2)	0.95468492	1.020134804
ENSDARG00000014215	cdh13	0.95030325	0.997214899
ENSDARG00000037478	CDH8	0.94743074	1.002028646
ENSDARG00000090614	BX005294.4	0.94697125	1.030584532
ENSDARG00000089562	BX957322.2	0.94650917	1.013223548
ENSDARG00000078898	pcdh7a	0.94598526	0.968654207
ENSDARG00000077996	CDH24 (2 of 2)	0.94435186	1.0096718
ENSDARG00000091043	pcdh2ab6	0.94287036	1.038341039
ENSDARG00000036121	pcdh10b	0.94147247	1.001641085
ENSDARG00000062720	pcdh1a	0.94049128	0.995317351
ENSDARG00000067692	CDH18 (1 of 2)	0.9393701	1.001919273
ENSDARG00000027041	pcdh17	0.93906712	1.002631778
ENSDARG00000063264	PCDH9	0.93491401	1.011713838

ENSDARG00000079640	pcdh2g16	0.93254535	1.028956493
ENSDARG00000015002	cdh4	0.93192655	0.986572104
ENSDARG00000073883	clstn3	0.92933325	0.96812777
ENSDARG00000079937	pcdh2g5	0.92930065	1.040329386
ENSDARG00000010684	CU915772.1	0.92664645	1.044601918
ENSDARG00000079424	CT573264.5	0.92578984	1.025192872
ENSDARG00000071889	pcdh2g8	0.92567154	1.017677281
ENSDARG00000076461	pcdh1g18	0.92448386	1.013495562
ENSDARG00000089593	pcdh2ab11	0.92442931	1.081910785
ENSDARG00000077343	CT573264.2	0.92439966	1.007783568
ENSDARG00000076707	pcdh2g29	0.92432192	1.016904131
ENSDARG00000058875	pcdh2ac	0.92271158	0.999411367
ENSDARG00000061371	cdh18	0.92186093	1.007141149
ENSDARG00000075978	pcdh1gc5	0.92179439	1.021230489
ENSDARG00000074738	pcdh2g9	0.92117006	1.026385513
ENSDARG00000071890	pcdh2g7	0.92000759	1.044900978
ENSDARG00000086307	CR847851.1	0.91975801	1.053116715
ENSDARG00000090748	pcdh2ab9	0.9136902	1.043222668
ENSDARG00000091924	pcdh1a3	0.91289555	0.971663508
ENSDARG00000071891	pcdh2g6	0.91107869	1.046691315
ENSDARG00000043951	pcdh1g9	0.9089895	1.0174073
ENSDARG00000073755	pcdh1g22	0.90835506	1.020525641
ENSDARG00000088475	pcdh1gb9	0.90430472	1.016772813
ENSDARG00000078940	pcdh2g12	0.90323167	1.01595193
ENSDARG00000071904	pcdh2ab1	0.90288208	1.04685759
ENSDARG00000091395	CABZ01067945.1	0.90254863	1.003201934
ENSDARG00000088724	pcdh2ab10	0.90196094	1.01224514
ENSDARG00000087974	pcdh2ab3	0.90037477	1.017588922
ENSDARG00000088053	CR847851.3	0.89998212	1.076413693
ENSDARG00000078400	BX005294.3	0.89804356	1.026523736
ENSDARG00000086619	CR847851.2	0.89753577	1.088564338
ENSDARG00000076107	pcdh1gb9	0.89503401	1.042119653
ENSDARG00000078226	CDH12 (2 of 2)	0.89385193	1.018187538
ENSDARG00000088522	CR847851.5	0.89308955	1.091425554
ENSDARG00000037286	DCHS2	0.8923997	1.008159164
ENSDARG00000042586	PCDH8	0.89044839	0.986307413
ENSDARG00000076459	pcdhb	0.88931256	0.971096819
ENSDARG00000071905	pcdh2ab3	0.88754309	1.051433279
ENSDARG00000075260	CT573264.1	0.88413008	1.019786473
ENSDARG00000061909	CU855896.1	0.88276655	0.985840525
ENSDARG00000089297	CR847851.8	0.88162566	1.070734704

ENSDARG00000089874	pcdh2ab8	0.88139414	1.071984548
ENSDARG00000088136	CR847851.4	0.8802254	1.035509734
ENSDARG00000077867	CT573264.3	0.87933268	0.999591888
ENSDARG00000088587	CR847851.5	0.87902786	1.102082902
ENSDARG00000055843	cdh10	0.87192884	1.006616443
ENSDARG00000037695	pcdh2aa1	0.86646396	1.017812728
ENSDARG00000077023	CU855896.2	0.86523883	0.981751194
ENSDARG00000077755	zgc:162322	0.86464887	1.009955722
ENSDARG00000087698	AL929558.3	0.86394593	1.025060636
ENSDARG00000086485	AL929558.1	0.85528118	0.995347194
ENSDARG00000073973	pcdh2aa3	0.8548708	1.011555708
ENSDARG00000006467	pcdh8	0.83656495	1.007976907
ENSDARG00000043961	PCDH10	0.83631734	1.010869269
ENSDARG00000087958	pcdh2aa15	0.83098476	1.013656661
ENSDARG00000076583	pcdh2aa1	0.82576274	0.990707194
ENSDARG00000088752	pcdh2ab7	0.81761549	0.958570497
ENSDARG00000088380	AL929558.4	0.78406254	0.983555815
ENSDARG00000088418	AL929558.5	0.76717993	1.011396427
ENSDARG00000086231	CT573264.4	0.76684408	0.956704381

Supplementary Table 1. Morphant expression values for homophilic cell adhesion genes. Microarray expression fold-changes in *pitx2ab* and *tbx5a* morphants are shown for genes annotated as being involved in 'homophilic cell adhesion' (GO:0007156).
